# Microsatellite markers used for genome-wide association mapping of partial resistance to *Sclerotinia sclerotiorum* in a world collection of *Brassica napus*

**DOI:** 10.1007/s11032-016-0496-5

**Published:** 2016-06-01

**Authors:** Sanjaya Gyawali, Myrtle Harrington, Jonathan Durkin, Kyla Horner, Isobel A. P. Parkin, Dwayne D. Hegedus, Diana Bekkaoui, Lone Buchwaldt

**Affiliations:** Agriculture and Agri-Food Canada, 107 Science Place, Saskatoon, SK S7N0X2 Canada; International Center for Agricultural Research in the Dry Areas (ICARDA), Rabat, Morocco

**Keywords:** Genome-wide association mapping, *Brassica napus*, Oilseed rape, Canola, Simple sequence repeats, SSR, Stem rot, *Sclerotinia sclerotiorum*

## Abstract

The fungal pathogen *Sclerotinia sclerotiorum* causes stem rot of oilseed rape (*Brassica napus*) worldwide. In preparation for genome-wide association mapping (GWAM) of sclerotinia resistance in *B. napus*, 152 accessions from diverse geographical regions were screened with a single Canadian isolate, #321. Plants were inoculated by attaching mycelium plugs to the main stem at full flower. Lesion lengths measured 7, 14 and 21 days after inoculation were used to calculate the area under the disease progress curve (AUDPC). Depth of penetration was noted and used to calculate percent soft and collapsed lesions (% s + c). The two disease traits were highly correlated (*r* = 0.93). Partially resistant accessions (AUDPC <7 and % s + c <2) were identified primarily from South Korea and Japan with a few from Pakistan, China and Europe. Genotyping of accessions with 84 simple sequence repeat markers provided 690 polymorphic loci for GWAM. The general linear model in TASSEL best fitted the data when adjusted for population structure (STRUCTURE), GLM + Q. After correction for positive false discovery rate, 34 loci were significantly associated with both disease traits of which 21 alleles contributed to resistance, while the remaining enhanced susceptibility. The phenotypic variation explained by the loci ranged from 6 to 25 %. Five loci mapped to published quantitative trait loci conferring sclerotinia resistance in Chinese lines.

**Electronic supplementary material:**

The online version of this article (doi:10.1007/s11032-016-0496-5) contains supplementary material, which is available to authorized users.

## Introduction

*Brassica napus* L. and other *Brassica* species are grown worldwide. In temperate regions, *B. napus* varieties low in glucosinolates and erucic acid (canola) provide vegetable oil for human consumption, while the protein-rich meal is added to animal feed rations. In some parts of the world, *B. napus* is traditionally grown as a leafy vegetable, whereas biofuel production is a relative new use for this crop in other regions (Abbadi and Leckband 2011).

The plant pathogenic fungus *Sclerotinia sclerotiorum* (Lib.) de Bary has a wide host range including numerous dicot crop species, such as oilseed rape where the disease causes symptoms known as stem rot or white mold. The pathogen forms sclerotia (resting bodies of mycelium) that can survive in the soil for several years. The sclerotia germinate and produce apothecia from which ascospores are dispersed within the crop. Spores infect plants by first colonizing senescent petals adhering to stems and leaves followed by direct penetration of the plant’s epidermis. Yield loss results predominantly from colonization of the main stem which blocks transport of water and nutrients to the developing seeds. It has been estimated that for each 1 % infected plants there is a 0.5–0.7 % yield reduction (Morral et al. 1984; Del Río et al. 2007; Kirkegaard et al. 2006; Koch et al. 2007). Fungicides have to be applied prophylactically which makes it inherently difficult to obtain consistent economical returns. Consequently, genetic resistance for controlling *S. sclerotiorum* is desirable both economically and environmentally.

Plant breeders in China were the first to develop partially resistant varieties, such as Zhongyou 821 and Zhongshuang 9 (Li et al. 1999; Wang et al. 2004). Quantitative resistance loci (QTL) were subsequently mapped in several Chinese cultivars and breeding lines (Zhao and Meng 2003; Zhao et al. 2006; Yin et al. 2010; Wu et al. 2013), one European cultivar (Wei et al. 2014) and a wild Brassica species *B. incana* (Mei et al. 2013) all demonstrating that sclerotinia resistance is a complex trait. However, the level of resistance was relatively low and prompted researchers to screen more *B. napus* varieties, breeding lines and land races (Zhao et al. 2004; Bradley et al. 2006), other brassicas (Li et al. 2007; Mei et al. 2011) and crucifer species (Uloth et al. 2013). Introgression of resistance from distantly related species into oilseed rape has been undertaken (Garg et al. 2010; Navabi et al. 2010; Ding et al. 2013), but is hampered by linkage drag of undesirable traits, varying levels of sterility and occasionally insurmountable crossing barriers.

To date, mapping of sclerotinia resistance loci has been carried out in populations derived from a cross between susceptible and resistant parents. However, marker–trait associations can also be identified in a collection of unrelated lines using the genome-wide association mapping (GWAM) approach. This method exploits the non-random association between phenotypic traits and molecular markers in genetically diverse germplasm in which random recombination events have accumulated over millennia (Flint-Garcia et al. 2003; Rafalski 2010). In *B. napus*, GWAM has so far been used to map glucosinolate content (Hasan et al. 2008), oil content (Zou et al. 2010), phenolic compounds (Rezaeizad et al. 2011) and several seed traits (Honsdorf et al. 2010). Jestin et al. (2011) used association mapping to identify loci conferring quantitative resistance to the fungal pathogen *Leptosphaeria maculans* causing blackleg in oilseed rape. Recently, GWAM was employed to map resistance to sclerotinia in *B. napus*, sunflower and soybean (Wei et al. 2015; Fusari et al. 2012; Bastien et al. 2014). Wei et al. (2015) were able to identify three loci associated with resistance in primarily Chinese *B. napus* lines using a disease screening method that involved wounding of the plant tissue. We set out to screen a more diverse collection of *B. napus* germplasm from various parts of the world using an inoculation method that resembles natural infection. The objectives were to identify new sources of sclerotinia resistance and to utilize the GWAM approach at various stringencies to demarcate a larger set of molecular markers contributing to resistance.

## Materials and methods

### Plant material

Initially, more than 400 *B. napus* accessions from diverse geographical regions of the world were screened for resistance to *S. sclerotiorum*. A subset of 152 accessions was selected consisting of lines with a consistent sclerotinia phenotype among single plants including the most resistant and susceptible lines. These lines represented three important productions areas of the world, Canada, China and Europe, as well as South Korea and Japan which are rarely examined in other studies. A large number of lines had been received by scientists at the Saskatoon Research Station (SRS prefix) over the years (Table S1). Seeds of other lines were obtained from three gene banks, the Plant Gene Resources of Canada (CN), the Nordic Gene Bank (NGB) and the Gene Bank in Prague, Czechoslovakia (15O0). The countries with number of accessions in brackets were as follows: Argentina (1), Australia (1), Canada (11), China (14), eleven countries in Europe (31), Japan (19), South Korea (58), Pakistan (15) and two of unknown origin (Table S1).

### Sclerotinia phenotyping

A single *S. sclerotiorum* isolate #321 was used for all inoculations. This isolate was collected in 1992 and represents a common clone identified in oilseed rape fields in the province of Alberta, Canada (Kohli et al. 1995). The aggressiveness of the isolate was maintained by inoculating the susceptible Canadian variety, Westar, every 5–6 months. Re-isolated cultures were stored either as mycelium at −80 °C in cryo-freezer solution (5 % skim milk in 20 % glycerol) or as sclerotia at 4 °C until needed for inoculation.

Seed of the 152 *B.napus* accessions was sown in a soilless mixture supplemented with slow-release fertilizer with one seed per 12.5-cm pot and five pots per line. The pots were placed randomly on a greenhouse bench in a 16-/8-h day/night cycle with light provided by high-pressure sodium lamps. The temperature was maintained between 21 and 24 °C during the day and 17–18 °C during the night. Pots were drip-irrigated and after 3 weeks fertilized weekly with NPK 20–20–20 (3 g/L). When plants were at full flower, the main stem was inoculated with *S. sclerotinia* as originally described by Buchwaldt et al. (2005) as follows. Isolate #321 from a stock culture was plated in 9-cm Petri dishes on glucose media (glucose 20 g, malic acid 3 g, NH_4_NO_3_ 2 g, KH_2_PO_4_ 1 g, NaOH 1 g, MgSO_4_7H_2_O 0.1 g, agar 20 g, distilled water 1000 mL) modified from Cruickshank (1983). Plates were incubated at 22/18 °C in a 16-/8-h day/night cycle with light provided by white fluorescent bulbs. After 4–6 days, and before hyphae reached the edge of the Petri dish, mycelium plugs (5–7 mm diameter) were cut from the actively growing margin. Each plug was placed on a 3 × 6 cm piece of Parafilm and attached to the main stem with mycelium facing the epidermis. Each stem was inoculated at two internodes with two un-inoculated internodes inbetween to avoid the developing lesions growing together. The length of each stem lesion was measured 7, 14, and 21 days after inoculation (dai). On these dates, the depth of penetration into the stem was assessed by slightly pressing each lesion and recording it as either firm, soft (stem not girdled) or collapsed (stem completely girdled). Percent soft plus collapsed lesions (% s + c) was calculated for each line. The area under the disease progress curve (AUDPC) was calculated using the lesion lengths from the three dates as follows $\sum_{n} [(L_{i} + \, L_{i + 1} /2)][(t_{i + 1} - \, t_{i} )] \, ,$ where *L*_*i*_ is lesion length on day *i*, *t* the number of days between observations and *n* the number of observations. The 152 *B. napus* accessions were screened in batches of 30 lines with the Canadian variety Westar as a susceptible control included in every test. Five plants of each accession were screened in one or two tests for a total of 10–20 stem lesions per line.

### Genotyping with SSR markers

Seeds of 152 *B. napus* lines were grown as described above, and leaf tissue was collected from single 4–5-week-old plants and immediately frozen in liquid nitrogen. The tissues were freeze-dried in a Freezone 6 dryer (Labconco Corp, KS, USA) for 48 h and then ground in a Mixture Mill 300 (Retsch Inc, PA, USA). DNA was extracted using a CTAB method, quantified using a NanoVue Plus spectrophotometer (GE Healthcare, NJ, USA) as per the manufacturer’s instruction, diluted to 10 ng/μL and kept at −20 °C until needed for genotyping.

Around 700 primer pairs flanking simple repeat DNA sequences in *B. napus* were developed at Agriculture and Agri-Food Canada beforehand, and the intervening DNA sequence of each locus was known. Eighty-four of these simple sequence repeat (SSR) markers were selected based on their polymorphism in selected *B. napus* lines, even distribution across the genome and high reproducibility in previous tests. A list of forward and reverse primers for the 84 SSRs were published previously (Gyawali et al. 2013). The forward primer (5′ end) of each SSR marker was labeled with either a 6-FAM (6-carboxyfluorescein), HEX (hexachloro-6-carboxyfluorescein) or TET (tetrachloro-fluorescein) fluorescent tag. DNA was amplified by PCR in 384-well plates using a C1000 thermal cycler (BioRad Life Sciences, CA, USA). Reactions were carried out in 12 µL volume containing 0.25 mM dNTPs, 1× PCR buffer (containing 15 mM MgCl_2_), 0.5 μM forward and reverse primer, 0.25 units *Taq* DNA polymerase and 20 ng genomic DNA. PCR was initiated with a hot start at 95 °C for 3 min, followed by 35 cycles of 94 °C for 30 s, 50 °C for 30 s and 68 °C for 1 min, with a final extension step of 68 °C for 5 min. Amplification products with different fluorescent tags were multiplexed and analyzed by capillary electrophoresis on a MegaBACE 1000 platform (GE Healthcare, NJ, USA). Data were analyzed using Fragment Profiler version 1.2 (GE Healthcare, NJ, USA). The SSR alleles were scored with GeneMapper v 3.7 (Applied Biosystems) according to their size using a 550 bp size standard included in all samples. Each peak was visually confirmed at least twice. SSR alleles were scored as dominant markers with 1 = present and 0 = absent, since *B. napus* is an allotetraploid species with two sub-genomes, AACC (2*n* = 38), and because homozygous and heterozygous loci could not be differentiated. A total of 1359 microsatellite alleles were identified, which was reduced to 690 after filtering for low allele frequencies of <5 %.

### Analysis of population structure Q and Δ*K*

In preparation for GWAM, both population structure (Q) and kinship (K) in the *B. napus* collection were analyzed to reduce the possibility of false positive trait–marker associations. Population structure was analyzed using the STRUCTURE version 2.3 software program (Evanno et al. 2005). It uses a Bayesian clustering method to obtain a Q-matrix that describes the percentage of parentage for each genotype by placing individuals randomly into *k*-number of subpopulations. The set of 690 SSR scoring data was analyzed from *k* = 1 to *k* = 10 with five independent iterations for each value. The Markov Chain Monte Carlo model (MCMC) was used with admixture ancestry and independent allele frequency parameters as suggested by Pritchard and Wen (2004). To reduce the effect of the initial configuration, a burn-in period of 10^5^ iterations was selected followed by another 10^5^ iterations. STRUCTURE HARVESTER version 6.7 (Earl and vonHoldt 2012) was used to visualize the likelihood of each number of subpopulations, Δ*K*, which is a modification of the kinship value *K*, according to the method suggested by Evanno et al. (2005). The most likely number of subpopulations was selected, and the average of the five corresponding iterations resulted in a final Q value for each *B. napus* accession which was used to assign the lines to the different subpopulations (Table S1). The population structure was also examined by principal coordinate analysis (PCoA) in NtSYS version 2.2 using the DECENTER and EIGEN functions with the first two coordinates plotted in a graph. Each *B. napus* accession was labelled with a letter for country of origin and consecutive numbers (Table S1).

### GWAM

The marker–trait relationship was analyzed with a general linear model (GLM) and the mixed linear model (MLM) in TASSEL version 3.0 (Bradbury et al. 2007). The mixed model approach was used as described in detail by Yu et al. (2006). Thus, two models were tested in order to obtain the best association between SSR alleles and the two disease traits, namely GLM + Q and MLM + Q + K, both followed by a correction for positive false discovery rate, pFDR. The matrices were combined as follows: Sclerotinia resistance = STRUCTURE [Q] + SSR marker + error, sclerotinia resistance = STRUCTURE [Q] + relative kinship [K] + SSR marker + error. The pFDR (*q* < 0.05) was applied in order to test the statistical significance of all detected markers (Storey 2002). The output data included percent phenotypic variation explained (*R*^2^) by each significant SSR allele and the additive effect, where negative values indicate the presence of the alleles that enhance resistance, and positive values indicate the presence of the alleles that enhance susceptibility.

### Co-location of SSR in GWAM and published QTL from linkage mapping

Possible co-location of SSR loci in the present study and published quantitative trait loci (QTLs) conferring sclerotinia resistance was assessed using the physical chromosome location of markers. The full DNA sequence of all SSR markers in the present study was known. These sequences were queried against the *B. napus* genome sequence in the Genoscope database (http://www.genoscope.cns.fr/brassicanapus) (Chalhoub et al. 2014) using the default BLAT (BLAST-like alignment tool) algorithm thereby obtaining the chromosome location measured in nucleotides (Kent 2002). DNA sequence information was obtained for 220 markers underlying 65 QTLs in five publications, Zhao and Meng (2003), Zhao et al. (2006), Wu et al. (2013), Wei et al. (2014) and Mei et al. (2013). The markers consisted of either sequenced RFLP probes or forward and reverse primers of SSR and AFLP markers, ranging from 15 to 28 nucleotides in length. The RFLP sequences were queried directly against the *B. napus* genome using the BLAT algorithm as described above. Since the BLAT algorithm is unreliable with sequences shorter than 33 nucleotides, the SSR and AFLP primer sequences were queried against the *B. napus* genome sequence in the Brassica database (BRAD) (http://brassicadb.org/brad/index.php) using the BLASTn program. If no hits were recorded, the *B. rapa* and *B. oleracea* genomes were queried. The resulting intervening DNA sequences were subsequently used to query the *B. napus* genome in Genoscope as described above. In most cases, markers mapped to the expected chromosome based on the published information, and the most likely nucleotide location was selected based on the highest BLAT score (>100) and identity (>90 %), while hits to homologous chromosomes were discounted. Only in a few cases was the hit entirely to an alternate chromosome. SSR loci contributing to sclerotinia resistance in the present study were considered close to previously published markers if located within 500,000 nucleotides of the QTL peak, or in cases when the peak marker did not map to the expected chromosome, between the closest pair of flanking markers. The limit of 500 kb flanking the QTL peak was a conservative value selected based on the median of previously published estimates of linkage disequilibrium (LD) decay in *B. napus* (Qian et al. 2014).

## Results

### Partially resistant accessions

**Fig. 1:**
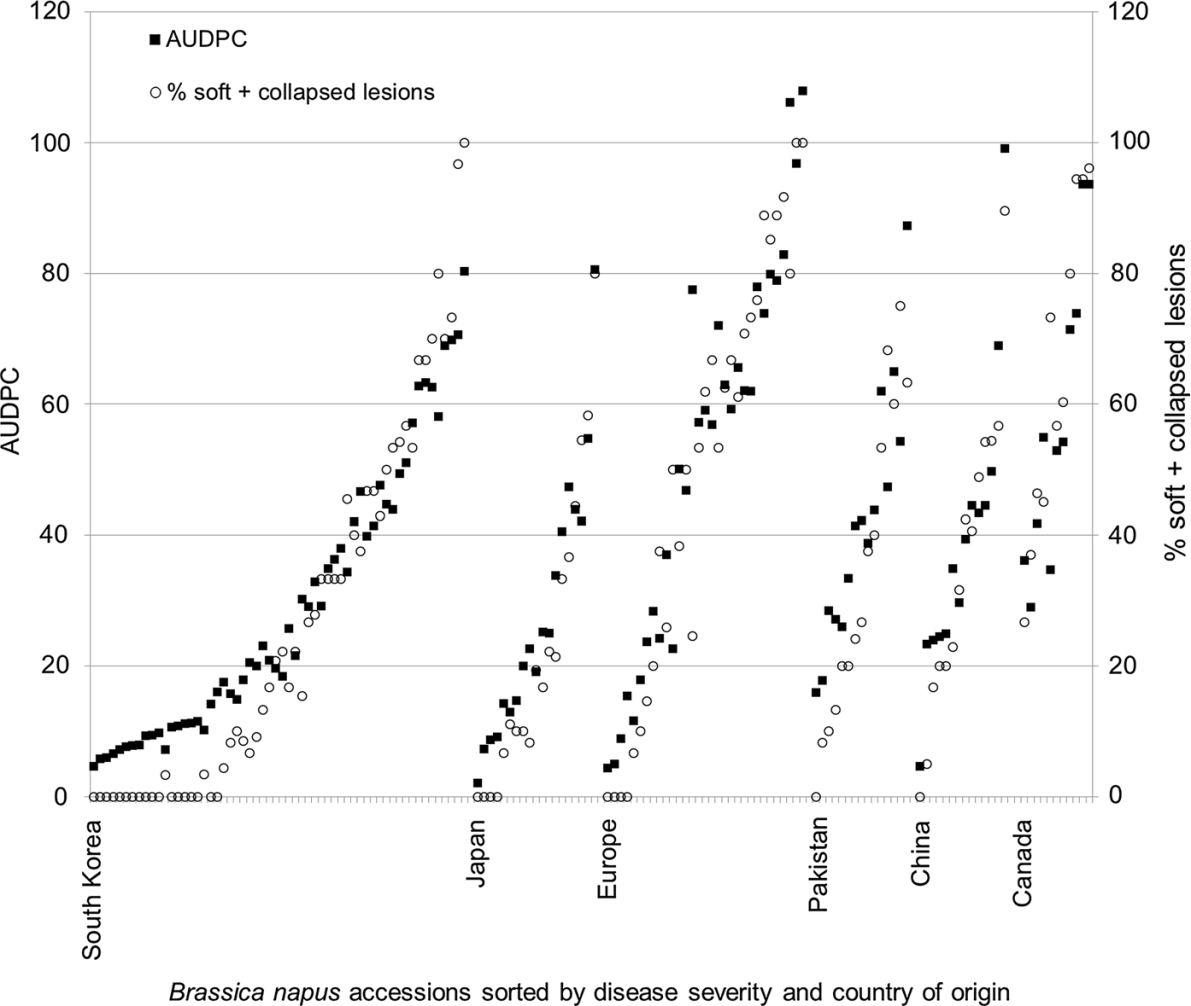
Results from screening a world collection of 152 *B. napus* accessions for resistance to the fungal pathogen *S. sclerotiorum*

Disease symptoms caused by *S. sclerotiorum* on the 152 *B. napus* accessions ranged from 2.1 to 107.9 AUDPC and from 0 to 100 % soft + collapsed lesions (Fig. 1; Table S1). A high positive correlation was found between the two disease traits by regression analysis, *r* = 0.93 (*P* < 0.001). The following accessions had the highest level of partial resistance to *S. sclerotiorum* isolate #321: Buk Wuk 3, 12, 13 and 20, Kuju 18, 19, 22, 27, 35 and 40, Dong Hae 3 and 6 from South Korea; Norin 16, Tokiwa natane, Kinki 22 and 30 from Japan; Tzuyechein 32 from China; SRS1632 from Poland and Tanto from France (Table S1). Based on a cutoff of AUDPC <10 and % s + c <10, the frequency of partially resistant lines was South Korea 19 %, Japan 21 %, Europe 10 %, China 7 % and Pakistan 6 %, while none of the Canadian lines were partially resistant. The data set used for GWAM was therefore somewhat biased for resistant lines originating from the neighboring countries, South Korea and Japan (Fig. 1).

### Subpopulations and kinship

**Fig. 2:**
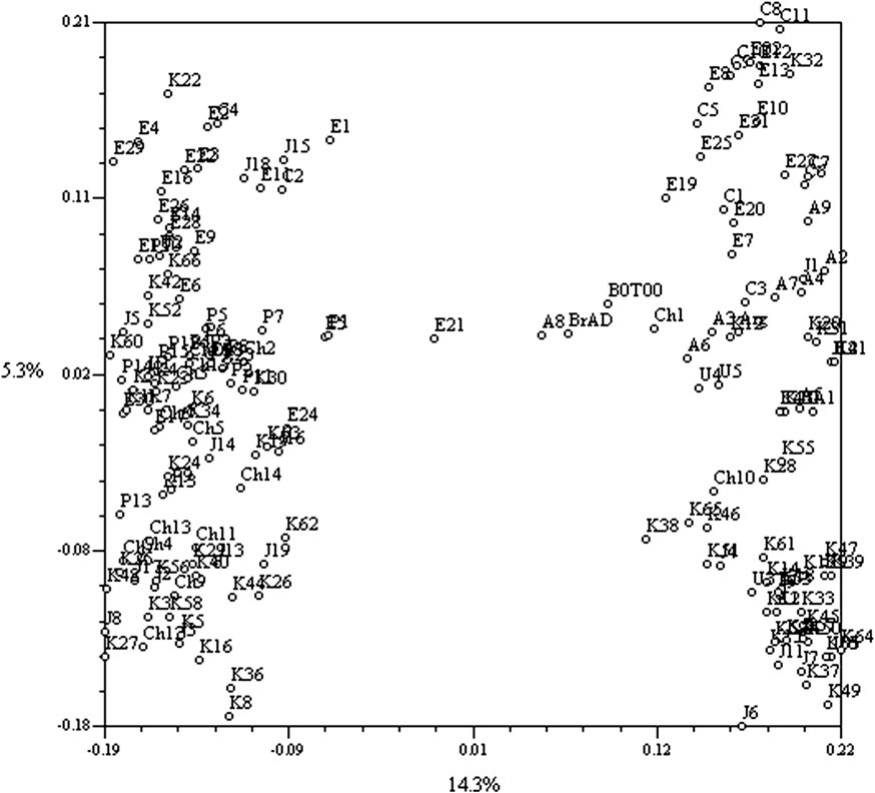
Principal coordinate plot of the first three variables based on polymorphism in simple sequence repeat (SSR) in a world collection of 152 *B. napus* accessions. The group on the *left*-hand side consists primarily of lines with semi-winter-type growth habit and the group on the *right*-hand side is mostly spring types

Analysis of population structure (Q) based on the clustering method in STRUCTURE, the ad hoc estimation of Δ*K* and PCoA all established the presence of two genetically distinct subpopulations. The result from PCoA is shown in Fig. 2. Based on a threshold of Q < 0.85 membership probability, 55 % (84) accessions belonged to subpopulation one (SP1), 38 % (58) to subpopulation two (SP2), while only 7 % (10) were placed in an admixed group (AD) (Table S1). Semi-winter-type accessions from China and Pakistan were unique to SP1, while all Canadian accessions were unique to SP2. Accessions from Europe, Japan and South Korea consisted of both semi-winter and spring types.

### SSR markers associated with sclerotinia resistance

Rare SSR alleles, which were identified in <5 % of the 152 *B. napus* lines, were eliminated to reduce detection of false marker–trait associations in subsequent data analysis. The allele frequency of the remaining 690 SSRs ranged from 5.3 to 94.5 % (Table 1). The GLM + Q model resulted in identification of 43 SSR loci significantly associated with resistance to *S. sclerotiorum* of which 34 were shared by both disease traits, AUDPC and % s + c. The *P* values of the remaining nine markers were low for one or the other trait (Table 1). The phenotypic effect (*R*^2^) explained by each marker ranged from 6 to 25 % for both traits, and the additive effect ranged from 18 to 41 % (Table 1). Of the 34 common loci, the additive effect was negative for 21 loci and positive for 13 loci indicating that their presence enhanced resistance and susceptibility, respectively.

**Table 1:** Simple sequence repeat (SSR) markers associated with resistance to *S. sclerotiorum* in *B. napus* identified by genome-wide association mapping in the GLM + Q model followed by a test of positive false discovery rate (*q*)

SSR marker allele	Linkage group (cM)^a^	Physical position^b^	Alleles		Area under the disease progress curve^c^			% Soft + collapsed lesions		
			Absent	Present	*P* value	*R* ^2^	Additive effect^d^	*P* value^e^	*R* ^2^	Additive effect
SR12355_213	A1: 79.6	A1: 22,709,214	49	82	2.8E−04*^,b^	9.8	−16.0			
SN3761_179	A2: 33.3	A2: 191,624	91	39	0.0016			4.9E−04*	9.2	20.2
SR0612_466	A2: 69.1	A2: 2,208,155	70	57	6.6E−05**	12.1	−17.1	4.0E−04*	9.6	−18.4
SR12015_304	na	A3: 9,607,501	41	90	5.4E−06**	15.0	20.2	1.2E−04**	10.9	20.7
SN0569_188	A3: 63.8	A3: 13,046,378	102	28				7.1E−04*	8.7	26.0
SR0536_237	A3: 93.5	A3: 23,044,269	109	19	9.2E−04*	8.4	−20.1	1.2E−05*	14.2	−31.5
SN13034_133	A4: 20.7	A4: 7,856,881	65	66	9.0E−06**	14.3	−18.4	1.8E−05**	13.4	−21.4
SN4276_480	A5: 45.2	A5: 4,793,850	91	39	9.1E−05**	11.4	−18.0	8.3E−05**	11.5	−21.9
SR12348I_180	A5: 18.8	A5: 8,755,826	108	22	2.1E−05**	13.3	−24.1	2.8E−04*	9.9	−25.0
SN1914_326	A6: 47.3	na	67	63	1.9E−08**	22.1	−22.8	3.5E−09**	24.1	−28.7
SN11795R_86	A6: 10.8	A6: 1,810,358 C6: 8,058,522	83	47	9.3E−08**	20.2	22.7	6.3E−08**	20.6	27.8
SN1914J_250	A6: 47.3	A6: 6,240,231	46	84	7.9E−05**	11.6	17.3	2.4E−04*	10.1	19.4
SS1949_205	A6: 67.6	A6: 19,329,293	91	40	4.8E−04*	9.1	15.9	8.5E−04*	8.4	18.3
SN12508_220	A6: 69.9	A6: 20,236,736	93	36	3.5E−05**	12.7	19.5	1.3E−05**	14.0	24.5
SS2141_137	A7: 63.3	A7: 1,881,404	89	40	1.7E−06**	16.7	−23.2	7.3E−07**	17.7	−28.7
SR7223_481	A7: 32.1	A7: 14,440,743	100	30	5.1E−04*	9.1	−17.3	3.5E−04*	9.6	−21.4
SR12173_203	A7: 54.9	A7: 16,297,589	68	56	8.4E−04*	8.8	14.6	2.5E−04*	10.5	19.2
SS2288_235	C8: 64.2	A9: 11,306,600	122	7	7.9E−04*	8.6	−31.5	2.2E−04*	10.3	−41.4
SN3560_346	na	A10: 11,967,893	90	39	2.9E−05**	13.0	−19.9	1.3E−05**	14.0	−24.9
SORH62_283	na	A10: 15,400,691	98	33	1.9E−04*	10.3	−18.5	1.6E−05**	13.5	−25.5
SR11329I_302	na	C1: rand_659,665	92	39	4.9E−05**	12.1	18.4	8.2E−06**	14.4	24.2
SN12788_290	C1: 54.8	C1: 29,218,479	63	66	4.3E−06**	15.5	−19.3	8.9E−05**	11.5	−20.0
SR12095I_442	A2: 0.0	C2: 1,738,778	92	36	2.4E−04*	10.3	−18.2	7.7E−05**	11.8	−23.5
SR12040_178	na	C2: 31,573,592	72	56				4.9E−04*	9.3	−18.1
SN0218_405	C2: 51.7	C2: 39,285,406	93	33	1.5E−04*	11.1	18.2	1.4E−04*	11.1	22.1
SN3821_495	C3: 40.9	C3: 8,220,706	95	35	2.3E−04*	10.2	18.1	1.4E−04*	10.8	22.5
SORD79_127	C3: 47.9	C3: 13,142,903	66	64	1.9E−05**	13.4	17.8	2.0E−04*	10.3	18.8
SNRA56_319	C3: 46.6	C3: 15,610,433	100	31				1.9E−04*	10.3	−22.2
SN0569_371	A3: 63.8	C3: 19,294,001	112	18	9.0E−04*	8.3	20.3			
SN2179F_226	C3: 66.9	C3: 23,259,732	10	121	1.4E−06**	16.7	−37.2	4.2E−04*	9.3	−33.4
SR12324I_336	C3: 79.3	C3: 34,968,604	93	37	1.3E−05**	14.0	−20.3	3.8E−06**	15.5	−25.9
SR1157_487	C4: 19.4	C4: 2,598,847	81	48	2.3E−06**	16.3	20.3	9.2E−05**	11.5	20.5
SN12745_278	C4: 29.0	C4: 6,035,809	25	106	6.4E−04*	8.7	−18.2	6.9E−04*	8.6	−21.7
SN0857_287	na	C4: 6,035,809	89	42	0.0046	6.1	−12.8	4.4E−04*	9.2	−19.0
SN1944_308	C5: 29.9	C5: 10,903,693	74	52	3.7E−04*	9.8	15.2			
SN0866_278	C8: 83.4	C5: 22,635	112	18	0.0011			1.9E−04*	10.4	−27.1
SORH06_416	C6: 41.5 C6: 43.2	C5: 27,616,764	73	56	1.9E−07**	19.4	−22.0	1.5E−05*	13.8	−22.3
SR6083_413	C6: 32.8	C6: 1,792,933	118	9	9.7E−05**	11.5	−32.8	1.4E−04*	11.1	−38.7
SN11542_199	C8: 18.2	C8: 8,182,817	100	24	9.3E−04*	8.7	−18.2	5.5E−04*	9.4	−22.9
SR12348I_114	na	C8: 24,799,307	70	60	5.9E−05**	12.0	16.8	3.6E−05**	12.6	20.8
SN1944_280	C8: 83.8	C8: 38,176,584	94	32				2.7E−04*	10.2	−21.2
SR12386_286	A5: 52.5	C8: 31,566,379	116	14	3.6E−05**	12.6	27.8	1.9E−04*	10.4	30.4
SN2016_270	na	C9: 13,616,587	54	72	2.6E−09**	25.1	−24.4	1.0E−07**	20.6	−26.7

^a^Centimorgan (cM) distances of SSR markers according to the integrated map by Wang et al. (2011); the locus with the strongest sequence homology is shown; na = information not available

^b^Location in nucleotides identified by DNA sequence homology in Genoscope (Chalhoub et al. 2014) presented as the mid-point of the sequence regardless of overall SSR size

^c^AUDPC calculated from the stem lesion length measured 7, 14 and 21 days after inoculation

^d^Negative values specify markers contributing to resistance while positive values specify susceptibility

^e^* *q* < 0.05; ** *q* < 0.001

**Fig. 3:**
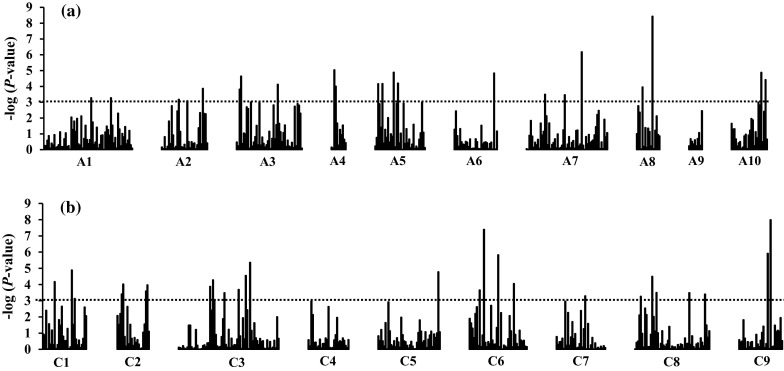
Graphic representation of negative log *P* values of SSR loci in *B. napus* associated with partial resistance to *S. sclerotiorum* by chromosome in the A and C genomes. The *dotted line* is the significance threshold level of *P* < 0.001 used to declare SSR loci significant in association mapping using the GLM + Q model. **a** Genome AA, **b** genome CC.

The density of loci varied between chromosomes A1–C9 as shown in the graph of negative log of probability in Fig. 3. Highest densities were found on chromosome A1, A3, A5, C3 and C6 and lowest on A4 and A9. SSR associated with sclerotinia resistance was equally distributed between the A and C sub-genomes (Table 1; Fig. 3). Only 44 and 20 % of the SSR markers associated with lesion length and % s + c, respectively, were significant in the MLM + Q + K model. Moreover, the positive false discovery rate (*q*) further declared all loci spurious except two.

### Co-location of SSR in GWAM and published QTL from linkage mapping

Of the 220 markers representing 65 previously published QTLs conferring sclerotinia resistance, 198 could be assigned to chromosome and nucleotide position in the *B. napus* genome. The remaining 22 markers were AFLP and SSRs without any sequence or primer information. Of 34 SSR loci obtained in the present study, 26 were associated with both AUDPC and % soft + collapsed sessions (Table 1). Of these 26 SSR, five were physically mapped within 500 kb of a published QTL peak marker or between the closest pair of flanking markers as follows: SR12095I on chromosome A2 in the present study mapped close to marker BGO153 and within the QTL region, BGO150-CB10022, identified by stem inoculation of *B. napus* line J7005 (Wu et al. 2013); SN12788 on C1 mapped close to SWUC150 identified by cut stem inoculation of *B. incana* line C01 (Mei et al. 2013), and within the QTL region, SWUC130-FITO083, identified by cut stem inoculation of *B. napus* cultivar Express from Germany (Wei et al. 2014); SR12040 on C2 mapped close to pW251 identified by petiole inoculation of *B. napus* line Huadbl2 (Zhao et al. 2006) and within QTL region, CB10316-Ol09A06, identified by cut stem inoculation of *B. napus* cultivar Express from Germany (Wei et al. 2014); SR12324 on C3 mapped close to FITO110 and SWUC455 identified by cut stem inoculation of *B. incana* line C01 (Mei et al. 2013); and SN12475 on C4 mapped within QTL region, pX105gE-pX116bH, identified by petiole inoculation of *B. napus* line Huadbl2 (Zhao et al. 2006).

## Discussion

The stem inoculation method used to phenotype the *B. napus* collection for resistance to *S. sclerotiorum* was first described by Buchwaldt et al. (2005). It was purposely designed to mimic the infection that causes the most serious yield loss in commercial fields. Moreover, inoculation using mycelium simulated the so-called infection cushions that are formed by fungal hyphae on senescent petals which adhere to the plants under wet weather conditions. The gradual spread of infection allowed rating of symptom development over several weeks. The relative difference among accessions became more obvious over time and was maintained up to maturity. The disease data collected 3 weeks after inoculation resulted in more significant marker-resistance associations than after 1 and 2 weeks (data not shown). Confidence in the association with the molecular markers identified in the present study was ensured by selecting 152 lines with consistent sclerotinia phenotype from a larger collection of 400 lines previously screened for resistance. Good separation in lesion length (measured as AUDPC) and penetration into the vascular tissue (measured as % soft and collapsed lesions) between resistant and susceptible lines also added to the confidence. The most resistant lines (AUDPC <20, % s + c <10) comprised 28 % of the mapping population, while the most susceptible lines (AUDPC >60, % s + c >70) comprised 20 %.

The advantage of disease rating 3 weeks after inoculation was also demonstrated by Li et al. (2007). The stem inoculation method has been adopted by others for screening of various brassica germplasm and crucifer species (Li et al. 2007; Garg et al. 2010; Navabi et al. 2010; Wu et al. 2013; Uloth et al. 2013). Other stem inoculation techniques have used insertion of toothpicks with mycelium into the stem (Zhao and Meng 2003; Yin et al. 2010), or placing cut stems in trays before inoculation (Ding et al. 2013; Mei et al. 2013; Wei et al. 2014). However, in the present study wounding of the plant was deliberately avoided, so as not to interfere with the expression of defense genes and molecular pathways in the epidermis, mesophyll and vascular bundles. Simpler and faster screening techniques have been employed, such as inoculation of cotyledons (Garg et al. 2008), detached leaves (Zhao and Meng 2003; Bradley et al. 2006; Wu et al. 2013) or placing of mycelium on the cut end of petioles of young plants (Zhao et al. 2004, 2006; Bradley et al. 2006). However, none of the methods resemble field infection, and the rapid deterioration of infected tissues in all cases necessitated assessment of symptoms after only 2–8 days.

In preparation for GWAM, more than 400 *B. napus* accessions were screened for resistance against *S. sclerotiorum*. Subsequently, the most resistant and susceptible lines within certain geographical regions were selected for a total of 152 lines. Since partial resistance was known to exist in germplasm from China, a large portion of accessions were selected from countries in Asia. However, it was not possible to create a balanced number of resistant and susceptible lines in all regions. Examination of genetic diversity and population structure of an almost identical collection of 173 *B. napus* accessions was published earlier, but without analysis of association to sclerotinia resistance (Gyawali et al. 2013). Both the previous and the present study showed the presence of two subpopulations primarily based on spring or winter growth habits as was discussed in detail previously. Subpopulations in *B. napus* are primarily based on growth habit, plant morphology related to crop utilization such as oilseed rape, swedes for fodder, and vegetables (Hasan et al. 2008; Chen et al. 2008; Bus et al. 2011; Rezaeizad et al. 2011).

As is apparent from GWAM studies in *B. napus* and other plant species, the best fitting model for association analysis is identified on a case by case basis, and the trade-off between dismissing false positives and accepting false negatives is somewhat arbitrary. In the present study, the GLM + Q model best fitted the data and identified 21 SSR alleles contributing to the reduction in lesion growth along the stem and depth of penetration into the stem explaining 6–25 % of the phenotypic variation, while the presence of 13 other SSR alleles contributed to susceptibility. In contrast, only two markers were significant in the MLM + Q + K model. Thus, this model increased the number of false negatives at the cost of reducing false positives, which should be avoided in an exploratory analysis like association mapping, as it is more important to identify significant alleles than avoid false positives (Honsdorf et al. 2010). False positives can be effectively controlled by applying a double statistics method that uses population structure matrix as a covariate in the regression model, followed by a correction for positive false discovery rate, pFDR (Hasan et al. 2008; Jestin et al. 2011; Rezaeizad et al. 2011). Jestin et al. (2011) identified 49 and 60 markers associated with blackleg resistance in *B. napus* using the GLM + Q and MLM + K models, respectively, while none of the markers remained significant when the MLM + Q + K model was applied. Yu et al. (2006) showed the MLM + Q + K model better described their data compared to GLM, GLM + Q and MLM + K, in accounting for false positives. Association mapping of sclerotinia resistance in sunflower was undertaken using DNA polymorphisms in selected defense genes. There, the MLM model was suitable and was not greatly affected by either Q or K matrices (Fusari et al. 2012). The MLM + Q + K model gave the best fit for mapping of sclerotinia resistance in soybean (Bastien et al. 2014).

An investigation was undertaken to determine whether the SSR loci identified by GWAM in the current study coincided with published sclerotinia resistance QTLs identified by linkage analysis using biparental (resistant and susceptible), doubled haploid mapping populations. Zhao and Meng (2003) mapped sclerotinia resistance QTL in the Chinese line Ning RS-1 by inoculating detached leaves rated 2 dai and by inserting toothpicks with mycelium into stems rated 5 dai. Three QTLs for each method were identified explaining 13–23 % of the phenotype, but none were shared by the methods. Zhao et al. (2006) mapped QTLs in both Huadbl2 (China) and Major (France) by cut petiole inoculation rated 4–8 dai, which resulted in eight and one QTLs, respectively, explaining 6–22 % of the phenotype. Yin et al. (2010) mapped QTLs in the Chinese variety Zhongyou 821 using stem inoculation with three types of inoculum (mycelium grown on either PDA, toothpicks or petals) with symptoms rated 3–7 dai. Although up to ten QTLs were identified for each method explaining 10–31 %, none of the loci were shared. Wu et al. (2013) mapped QTLs in the Chinese line J7005 grown at three field locations. The stems were inoculated with mycelium plugs and lesion length rated 7 dai. Ten QTLs were identified explaining between 7 and 32 % of resistance, of which markers on chromosome A9 and C6 were consistently significant over test locations. Also, detached leaves were inoculated with mycelium plugs and lesions rated 2 dai, but the three resultant QTLs were different from the stem QTLs. Wei et al. (2015) mapped QTL in the European winter-type variety Express both under natural sclerotinia infection and by mycelium plug inoculation of stems placed in trays rated 3 dai. Six and five QTL were identified using the two methods explaining 5–20 %, and one QTL on chromosome C2 was shared. Li et al. (2015) produced an integrated map using 540 SSR markers from these five publications and determined the location of 35 sclerotinia QTLs using the *B. napus* genome sequence in Genoscope (Chalhoub et al. 2014). Subsequently, they concluded that two sclerotinia QTL were conserved across studies, one on A9 (Wu et al. 2013; Wei et al. 2014) and C6 (Wu et al. 2013; Zhao et al. 2006), respectively. Examination of possible co-location of molecular markers underlying 65 QTLs in the same five publications with SSR loci in the present study showed that five loci (7.6 %) on A2, C1, C2, C3 and C4 were likely captured by GWAM. We conclude that despite differences in origin of *B. napus* accessions and phenotyping methods between studies, the overlapping map locations indicate that some of the defense mechanisms against *S. sclerotiorum* could be shared by different resistant *B. napus* lines. In our study, the GWA identified 21 loci conferring resistance to *S. sclerotiorum* and 13 loci conferring susceptibility mapped to 12 of the 19 *B. napus* chromosomes with almost equally distribution between the A and C genomes. Furthermore, none of the SSR markers were linked when the conservative 500-kb cutoff was applied. The GWA study conducted by Wei et al. (2015) identified three loci on A8, C3 and C6. Although the map location of individual resistance loci was determined using the same *B. napus* genome sequence in Genoscope (Chalhoub et al. 2014), none of the resistance loci were shared between the two GWA studies. The discrepancy can be explained by different inoculation and disease rating methodologies; the first study used inoculation of intact plant stems without wounding and measurement of stem lesion length over 21 days, whereas the second study used wounding and measurement of lesion length after only 3 days. Wounding of the stem surface may circumvent molecular or mechanical defense mechanisms during initial infection as also discussed by Wei et al. (2015). We conclude that differences in phenotyping method, *B. napus* germplasm, marker technology and association analysis all contribute to variation between the two studies.

Predictably, most markers associated with sclerotinia resistance in the present study were new, primarily because many resistance accessions were evaluated for the first time. Also, whereas the published QTL were detected in Chinese lines, the partially resistance accessions in the GWAM study originated mainly from South Korea (Buk Wuk 3, 12, 13 and 20; Kuju 18, 19, 22, 27, 35 and 40; Dong Hae 3 and 6) and Japan (Norin 16, Tokiwa natane, Kinki 22 and 30), and single accessions were identified from China (Tzuyechein 32), Poland (SRS1632) and France (Tanto). The new sources of resistant *B. napus* accessions identified in the present study are suitable starting points for development of improved oilseed rape varieties. However, screening with isolates from the area of planned utilization is prudent, since evidence of isolates with different levels of aggressiveness and host preference is accumulating (Ge et al. 2012; Taylor et al. 2015).

Since a relatively low coverage of the *B. napus* genome was achieved with 690 SSR loci in the present study, further research is underway to more accurately map loci conferring sclerotinia resistance including GWAM utilizing markers based on single nucleotide polymorphisms and QTL mapping in biparental, doubled haploid populations derived from some of the partially resistant lines from Pakistan, China, South Korea and Japan. In the future, this additional marker information will aid transfer of stem rot resistance to adapted oilseed rape varieties.

## Electronic supplementary material

Below is the link to the electronic supplementary material.

Supplementary material 1 (XLSX 27 kb)
